# Ocular Syphilis: A Case Report

**DOI:** 10.7759/cureus.23509

**Published:** 2022-03-26

**Authors:** Andreia M Teixeira, Elsa Meireles, Carla Pereira Fontes, Micaela Manuel

**Affiliations:** 1 Internal Medicine, Centro Hospitalar de Entre o Douro e Vouga, Santa Maria da Feira, PRT

**Keywords:** cryoglobulinemia, treponema pallidum, neurosyphilis, syphilis, uveitis

## Abstract

Neurosyphilis refers to the involvement of the central nervous system by *Treponema pallidum*. Ocular syphilis can present with a range of manifestations, uveitis being the most common, and it can occur at any stage of acquired syphilis. There are multiple tests available for the diagnosis of syphilis. Moreover, the treatment of syphilis depends upon the stage of the disease.

For years, the management of syphilitic uveitis was controversial among physicians, with several reviews debating whether ocular syphilis is a subtype of neurosyphilis. Recent recommendations state that ocular syphilis should be treated similarly to neurosyphilis, even with a normal liquor examination. Herein, we describe a case of a 57-year-old male patient who was diagnosed with ocular syphilis.

## Introduction

Syphilis is a sexually transmitted chronic disease caused by the spirochete *Treponema pallidum* and can be transmitted from mother to child (congenital syphilis) or can be acquired (acquired syphilis) [1].

The clinical course of acquired untreated syphilis can be divided into stages depending on clinical manifestations such as primary, secondary, and tertiary syphilis [2]. Latent infections (those lacking clinical manifestations) are detected by serologic testing and those acquired within the previous year are referred to as early latent syphilis, and the other cases are classified as late latent syphilis or latent syphilis of unknown duration [3].

Neurosyphilis is referred to as the invasion of the central nervous system by *Treponema pallidum* and it can be asymptomatic or present with vasculitis, stroke, dementia, meningitis, or psychosis [4]. Ocular syphilis can present with a range of manifestations, uveitis being the most common [5], and it can occur at any stage of acquired syphilis [1]. It can affect several parts of the eye presenting as anterior uveitis, posterior uveitis, panuveitis, retinitis, papillitis, and scleritis [6]. Syphilitic uveitis is a rare condition accounting for 1-2% of all uveitis [5], although the incidence of acquired syphilis is rising in several countries [7]. Treatment of syphilis depends upon the stage of the disease [8].

## Case presentation

A 57-year-old man with no prior conditions presented to the emergency department with complaints of left ocular pain, red eye, photophobia, and myodesopsias. He also complained of frequent oral ulcers without genital ulcers and maculopapular exanthema at the trunk, back, and superior limbs, sparing palms.

The evaluation showed stable vital signs, with normal blood pressure and heart rate and no focal neurological deficits or meningeal signs. Ocular examination demonstrated bilateral papillary oedema and left eye vitritis. A brain computed tomography scan ruled out parenchymal alterations that suggested vascular or space-occupying lesions. Lumbar punction with normal opening pressure revealed crystalline liquor, no pleocytosis with proteins, and glucose at the normal range. Skin biopsy unveiled leukocytoclastic vasculitis. Blood tests presented with normal hemogram and leucogram but revealed C4 consumption, cryoglobulinemia, and positivity to anticardiolipin IgM.

He was medicated with prednisolone 60 mg per day and evaluated in ambulatory medical consultation one week later. At the consultation, he maintained ocular complaints, although the rash and oral ulcers had resolved. He admitted to not taking the prescribed medication.

Further investigation for immunological and infectious diseases was initiated (Table 1) with positivity for Venereal Disease Research Laboratory (VDRL) (1:32 titer) and *Treponema* tests, both IgG and IgM. Due to ocular manifestations, the diagnosis of neurosyphilis was made and he was hospitalized and submitted to the traditional penicillin scheme for 14 days.

**Table 1:** Complementary exams of the patient. Anti-HBs: hepatitis B surface antibody; anti-HBc: hepatitis B core antibody; anti-HCV: hepatitis C antibody; EBNA: Epstein-Barr virus nuclear antigen; HBs antigen: hepatitis B surface antigen; HLA: human leukocyte antigen; HIV: human immunodeficiency virus; TPHA: *Treponema pallidum* haemagglutination assay; VCA: viral capsid antigen; VDRL: Venereal Disease Research Laboratory.

Exam	Patient result	Reference value
Haemoglobin	15.3 g/dL	13.0-17.0 g/dL
Platelets	290 x 10^9^/L	150-450 x 10^9^/L
Leukocytes	5.2 x 10^9^/L	4.0-11.0 x 10^9^/L
Prothrombin time	12.1 seconds	11.4 seconds
Activated prothrombin time	30.3 seconds	31.8 seconds
Sedimentation rate	13 mm	0-19 mm
Sodium	139.0 mmol/L	136.0-145.0 mmol/L
Potassium	4.7 mmol/L	3.5-5.1 mmol/L
Total calcium	9.6 mg/dL	8.4-10.2mg/dL
Albumin	4.4 g/dL	3.5-5.0 g/dL
Creatinine	0.8 mg/dL	0.7-1.3 mg/dL
Urea	39 mg/dL	18-55 mg/dL
Total proteins	8.1 g/dL	6.4-8.3 g/dL
Protein electrophoresis	Polyclonal increase of immunoglobulins	
Immunoglobulin A (IgA)	371 mg/dL	63-484 mg/dL
Immunoglobulin G (IgG)	1709 mg/dL	540-1822 mg/dL
Immunoglobulin M (IgM)	171 mg/dL	22-240 mg/dL
Antinuclear antibody (ANA)	Negative	-
Anti-neutrophil cytoplasmic antibody (ANCA)	Negative	-
Rheumatoid factor	<20 UI/mL	0-30 UI/mL
Anti-cardiolipin IgG	3.70	Negative if title <10
Anti-cardiolipin IgM	145.0	Positive if title >40
Cryoglobulins	Positive	-
C3 complement	126 mg/dL	82-185 mg/dL
C4 complement	9 mg/dL	15-53 mg/dL
HLA B51	Negative	-
HLA B27	Negative	-
B2-microglobulin	1.84 mg/L	<2.64 mg/dL
Anti-cytomegalovirus IgM	Negative	-
Anti-cytomegalovirus IgG	Positive	-
Epstein-Barr VCA IgG	Positive	-
Epstein-Barr EBNA IgG	Positive	-
Epstein-Barr EBNA IgM	Negative	-
HBs antigen	Negative	-
Anti-HBs	Negative	-
Anti-HBc	Negative	-
Anti-HCV	Negative	-
HIV (1 and 2)	Negative	-
Anti-herpes virus I IgG	Positive	-
Anti-herpes virus I IgM	Negative	-
Anti-herpes virus II IgG	Positive	-
Anti-herpes virus II IgM	Negative	-
VDRL	Positive (titer: 1/32)	-
TPHA	Positive	-

During hospitalization, the measurement of treponemal and non-treponemal test in the cerebrospinal fluid (CSF) came out positive, which reinforced our diagnosis and initial management.

During treatment, he was submitted to the ophthalmologic examination and papillary oedema improved. After discharge, he was evaluated closely and within four months after treatment, ocular alterations and the abnormalities in blood tests presented at admission were resolved completely, with no recurrence of symptoms.

## Discussion

The diagnosis of neurosyphilis is a challenge due to the variety of clinical signs and diagnostic techniques [4], and a negative VDRL test in the CSF does not rule out the diagnosis [1]. Many assays take time to be accessible and there is a considerable amount of false positive and false negative results [9], which increases the difficulty of diagnosis. Different tests are available for the diagnosis of syphilis and they can be divided into nonspecific tests such as VDRL and rapid plasma reagin (RPR), which quantify the amount of serum anticardiolipin antibodies, and *Treponema pallidum* specific tests such as fluorescent treponemal antibody absorption (FTA-ABS), *Treponema pallidum* particle agglutination (TPPA), and *Treponema pallidum* haemagglutination assay (TPHA), which measure the amount of serum antibodies specifically directed against treponemal antigens [5].

Furthermore, in our case, the patient presented with uveitis, oral ulcers, rash, and cryoglobulinemia, and low levels of C4 complement being the only readily available altered blood exams, which could mislead the diagnosis and prompt the investigation towards an extensive search for immunological or hematologic diseases rather than syphilis infection; therefore, clinical suspicion plays a major role [4]. It can mimic several diseases and courses with immunological phenomena such as leukocytoclastic vasculitis and cryoglobulinemia [10], which may lead to organ damage and the need for immunosuppressors or plasma exchange [10].

Implications of the optic disc with papillitis, optic neuritis, neuroretinitis, and panuveitis are consistent with an ocular manifestation of neurosyphilis [1]. Recent recommendations from* *the Centers for Disease Control and Preventionand European guidelines on the management of syphilis recommend that ocular syphilis should be treated similarly to neurosyphilis, even in the absence of abnormalities in the CSF [3,5]. The treatment of syphilis is done majorly with penicillin but the scheme depends upon the stage of the disease with a conventional regimen for neurosyphilis consisting of 12-24 million units of intravenous benzylpenicillin each day every four hours for a period of 10-21 days [1,11] or ceftriaxone 1-2 g daily either intramuscular (IM) or IV for 10-14 days as an alternative treatment for those with a history of penicillin allergy [3]. It is controversial whether corticosteroids or other immunosuppressants have efficacy when administrated in combination with antibacterial regimens [1].

## Conclusions

The prevalence of syphilis is rising and, therefore, its complications. Syphilis must be considered in a patient with uveitis, as it may be the first clinical manifestation of the disease, so its investigation must be pursued as prompt diagnosis and management impose prognostic value. Whether ocular involvement can be classified as neurosyphilis is still controversial. However, recent recommendations advise treatment as such in a patient with uveitis and positive treponemic or non-treponemic tests in the serum.
